# Sociological Medicine1An address delivered at the opening of the sixty-third annual session of the Medical Department of the University of Buffalo, September 28, 1908.

**Published:** 1908-11

**Authors:** Julius Ullman

**Affiliations:** Lecturer on Clinical Medicine, Medical Department University of Buffalo


### BUFFALO MEDICAL JOURNAL.

VOL. LXIV. NOVEMBER, 1908. NO. 4

### ORIGINAL COMMUNICATIONS.

### Sociological Medicine.[1]

By JULIUS ULLMAN, M. D.,
Lecturer on Clinical Medicine, Medical Department University of Buffalo.

TO you, who are about to begin the sixty-third regular session in furtherance of study and the acquisition of knowledge in your accepted vocation, I desire to welcome you to a return of the friendly relations of teacher and student. You enter the University for purposes of education in the science of medicine and your teachers and instructors return to you, after several months of rest and recreation, to disseminate through you the old and the newer truths based upon observation and experience, for the elevation of our chosen profession of medicine and for the preservation and uplifting of mankind.

Robert Louis Stevenson once wrote concerning the life of the Medical Man: "The physician is the flower of our civilisation, and when that stage of man is done with, and only remembered to be marveled at in history, he will be thought to have shared as little as any in the defects of the period, and most notably to have exhibited the virtues of the race." It is such a tribute to the honor, valor and value of our profession which makes us proud of our heritage and anxious to feed upon the ambition, so unselfish, to further the welfare of mankind, by not only combating the enemies which nature has in store for us, but to teach the doctrine of the prevention of disease and the fulfillment of good will and happiness to mankind. See to it that by your zeal, endeavor and industry, within these walls, you show a desire to emulate those who have labored wisely and well without regard to personal gain, and often unheralded, for the perfection and elevation of the science of medicine and have demonstrated that the triumphs of sanitary science, surgery and medical progress are among the grandest of civilisation!

We hear it often said that our profession is overcrowded. This is true in a relative sense, but it is equally true in all vocations of life and applies only to him who is indifferent, selfish,

1. An address delivered at the opening of the sixty-third annual session of the Medical Department of the University of Buffalo, September 28, 1908.

and who lacks industry and perseverance. There is a place for all of us directly in proportion to the exertion which we make. Do not, therefore, be discouraged, for success is not always measured by the accumulation of material worldly stores, but by the greater value of reputation "rich in the inheritance which it leaves, pious in the example which it testifies, pure, precious, and imperishable in the hope which it inspires."

A number of months ago at a testimonial dinner given Professor Koch, the great medical scientist, Andrew Carnegie who has gathered, who has given away, and who has still more money than any other one individual, said that he would give all his worldly possessions for the immaterial wealth of many of the physicians then before him, and confessed that his money was a poor substitute for the beneficent science of an intelligent medical man.

In spite of widespread skepticism, intolerance and pessimism, the practice of medicine holds greater and more wonderful possibilities today for the benefit of humanity than ever before.

Would you compare the relief of disease today with that of three centuries ago? According to Billings, in 1594 for every 1,000 children born at least fifteen and often forty mothers died. Nothing was known of the causes and nature of childbed fever which followed the visitation of the doctors and midwives like a pestilence,—a disease "which closed the eyes just opened upon a new world of life and happiness, bowed the strength of manhood to the dust and cast the helplessness of infants into strangers arms"

Have we not cause for optimism when we have conquered through efforts of legislation ophthalmia in the helpless newborn, which caused the eyelids to redden and swell and yellow matter to exude and in a few days or weeks shut out forever light from a soul? So also smallpox which before Jenner's discovery of vaccination killed 1-10 of all the people on the globe and disfigured nearly twice as many more. In Prussia, where vaccination is compulsory there is but one death to every 300,000 inhabitants. Diphtheria, which is now cured, killed unrelentlessly. Fevers and dysenteries decimated villages and prisons and but little was known of their differentiation.

The death-rate today is but one half what it was as late as fifty years ago. The average duration of life of a Roman citizen was thirty years. In Geneva during the 16th and 17th centuries, it was not more than twenty years. Today, it is between forty-five and fifty years. With Osler, we ask how much pain and suffering has been saved and prevented by the medical discoveries of the last fifty years—that is, the use of anesthetics, aseptic surgery, the control of epidemics and the beginnings of effective sanitation. It is difficult for the imagination to grasp the sum total of the good which has been accomplished in the alleviation of pain and suffering. The spirit of 1908 is a hopeful service in behalf of humanity.

In college work we have every reason to be hopeful when we compare the teaching of medicine today with what it was in the early part of the 19th century. The doctor in 1800 received all homage to what seemed to the layman in those days almost a supernatural superiority, a homage which to us seems somewhat absurd when we consider how he was educated to his work. The student was the pupil of his preceptor—a practitioner, who often had never seen a skeleton nor performed an autopsy. These men often were graduates of the University of Hard Knocks and thus had none of the advantages you are about to receive. We begin the sixty-third annual session with a history of an honorable career and remember those professors now dead whose names add luster to the histroy of medicine in the new world,—the elder Flint, White, Hamilton, Dalton, Miner, Moore and the elder Rochester.

America was formerly held in ridicule abroad for its medical courses in the colleges, and when you consider that the United States is said to have 48 per cent. of the medical schools of the world, it is not surprising that though among the number may be mentioned some of the best institutions of their kind, there still remains a goodly number which will soon be eliminated following the Huxley law of the survival of the fittest. Great progress has been made of raising not only the premedical requirments by the respective state boards but also in the increase of years of attendance and the lengthening of the semesters.

Our University, though not enriched by large endowments, has nevertheless kept well in the foreground of advance, as evidenced by the excellent work of its alumni in practice throughout the United States and by the high standing of its more recent graduates before the state examining boards. During the past year through the untiring efforts of our Vice-chancellor, we have witnessed the first step taken toward the founding of a Greater University of Buffalo. What was thought a phantasy will soon become a happy realisation.

Men formerly entered the ranks of the profession of medicine for selfish purposes of self aggrandisement. Medicine offered an easier livelihood than trades and for this reason our noble calling has in the past been open to sharp satirical attack. There has been an evolution in the ethics of our profession and an effort at a higher morale and in this evolution you must take part by every act and precept.

The proper study of mankind was man and he was studied even to the minutest individuality of the cell, thanks to the work of the immortal Virchow. The medicine of the past concerned itself with the diagnosis, pathology and treatment of disease in the individual genus, man. We practised individualism in our art and forgot to heed the study of collectivism. We studied the pathological processes in man but failed to pursue the study of pathological processes in mankind. Charlotte Gilman states that we are subject to a universal law—namely, that a power to feel implies a power to act. What feels can move and thus we are grown socially conscious. We can treat social conditions when we have mastered their pathology.

Primitive man was most affected by physical conditions. He had to adjust himself to the exigencies of climate, soil and animal competition. Modern man has to adjust himself to social conditions. He is most affected by governments, religion, economic systems and general customs. Like prehistoric man we are still decimated by wild beasts which, thanks to Loewnschoek, Koch and others, we are able to study under the powerful lens of the microscope, but our human progress is still retarded by social pathology,—ignorance, poverty, crime and disease. Society has long since mastered the difficulties of adjustment with physical conditions but cannot arrange its intersocial conditions. These social pathological conditions are not all essential to our environment, but are due to economic errors and superstition.

Benjamin Andrews (The Duty of Public Spirit) says, "We need a larger, heartier recognition of men's dependence upon one another and of the moral and religious duties springing out of this close relationship. No man can live to himself so none ought to wish to do so." G. Lloyd Morgan states that those animals living in close association, the animals which take greatest pleasure in society, best escape various dangers; while those which cared least for their comrades perish in greatest numbers. "We are one of another and should so regard ourselves. If one suffers, all are hurt. The true weal of one is a blessing to the rest."

In conditions of environment, people live wholly unlike,—such as wealth, personal attributes of mind or body, financial, social and political opportunities. In the deeper things such as conscience and consciousness, emotions of hope, ambition, love, pride and contentment, joy or sorrow and those elements of rest and unrest, approximate satisfaction or endless longing and in all that constitutes humanity, we are subject to the same laws. Sociology is, then, the study of social relations, the mutual dependence, the helpfulness of true reciprocal duties and obligations of men toward men. In such a study, it is self-apparent how important becomes the activity of the medical profession.

### MEDICAL SOCIOLOGY.

I have, therefore, chosen as the subject of my discourse to you this evening, Medical Sociology.

As anatomy, biology, physiology and pathology are the foundations of your studies they are but a correlation of other sciences belonging to our calling. If you would be proficient therapeutists, you require knowledge of pharmacy and must needs know botony. If you desire knowledge in malaria you must know not only the plasmodium of Leveran which produces it, but the life history of his host, the mosquito, the anopheles. If you would release mankind from many of the evils of which it now suffers, you must study the conditions under which these evils exist and knowing them by collective as opposed to individualistic effort, remedy them.

We have had sociologists in medicine before society demanded a special study of itself and its conditions. Moses was a statesman and sociologist, the wisdom of whose teachings are too often disregarded. Pinel when he loosened the fetters and iron bonds which bound those bereft of reason to the unsanitary madhouses, and treated them not as low and degraded criminals possessed of evil spirits, was a sociologist. Jenner, when he through vaccination gave life in place of death, Morton and Simpson in introducing anesthesia, Koch, Pasteur and Behring in bacteriology, Lister in demonstrating aseptic surgery, Reed and Carrol in showing the method of dissemination of yellow fever and Colonel Gorgas in demonstrating that it is possible to make Havana, and the Panama Canal zone safe for human life, are sociologists of whom we can be proud. Thus the physician has assisted in sociologic progress for by his efforts the world has advanced in the knowledge of the real nature of disease and its rational treatment.

Doctor Kime states that this is a sociologic age and we as a nation have reached that stage in evolution in which sociology is an important factor in our development. We must recognise sociological principles or become diseased physically, morally and mentally as we increase in wealth, population, and age. We are no longer a rural people but with the progress of inventions in steam, electricity, hydraulics, and machinery, there has been a wonderful increase in the resources of the country with its consequence of an entire revolution and rearrangement of our lives; thus the rapid intercommunication of travel between the most diverse countries and peoples has brought into prominence problems which are yet to be solved.

Sir James Paget defines as the healthiest nation the one composed of healthy men, who live long and vigorous lives, who in every part of their life wheresoever and whatever it may be, do the largest amount of the best work that they can and when they die leave healthy offspring. If then, we are to do the best work, we must have health and it is here where the science of medicine is the cog for the machinery of life well done. A community does not know the meaning of sickness except perhaps in an epidemic when all lives are threatened, nor does a community appreciate the value of work until it is lost.

Mr. Sutton, the Actuary of the Registry of Friendly Societies of England, has returns of many years of sickness and mortality among members of a very large number of societies and there is recorded the number of days men receive money when "off work." He deduces the average number of days' sickness per member per annum is 1 1|3 to 1 3|4 weeks for the male population and for the female population a fraction more. Among the males, then, a loss of 9,692.505 weeks per year and among the female population 10 592.761 weeks, hence for the entire population between fifteen and sixty-five years 20,000,000 weeks are lost by sickness. These people in good health earn good wages and salaries and do not include invalids, insane, imbeciles, idiots or habitual drunkards. These figures, then represent about 1|40 of the work done by the entire population between fifteen and sixty-five or in money 11,000,000 pounds sterling per week. Let us for a moment examine the loss occasioned by a preventable disease like typhoid fever. In England, typhoid with a mortality of about 15 per cent. destroys 4,000 lives; 23,000 recover. The average length of the disease is 10 weeks, so that 23,000 weeks are lost in work. This does not include the loss of happiness and welfare due to sickness.

Again, according to Hunter, the cost to society to prepare a man for usefulness is $1,500. This is a grant from parent or state to a child which he is expected to repay. If in the state of New York, for instance, 15,000 die each year from a preventable disease like tuberculosis, there is a loss of $22,500,000 and with the cost of medicines, nursing, loss of labor, another $16,000,000 may be added, so that it is computed that in the United States tuberculosis occasions a loss of $330,000,000 annually and that the loss from preventable diseases reaches the stupendous sum of $600,000,000 annually. Again, consider the loss of work throughout life from the sickness in early childhood. How many through disease in childhood brought on through no fault of their own are made more susceptible to diseases in later life, are crippled or permanently disabled and are made recruits for the army of poverty?

Blackmar, in Elements of Sociology, says the productivity of a community is determined by the labor power and the elements which tend to labor as a means to wealth are the strength, the ability, the happiness, and above all the physical well-being of the laborer.

As a nation we have as yet not attained the knowledge that wealth must be secondary to health. We are as yet not cognisant that the profits of the individual should be secondary to the life, welfare, and prosperity of the great masses of the people and that human life must be placed before property. Suppose that a study of the economics of human life just referred to were made in reference to crops or meat supply. (Thousands have been spent in stamping out cholera among swine, but not a dollar was ever voted to eradicate pneumonia in human). What a national calamity would be the cry! What rigid steps would be demanded of the government to stop the loss! There is an economy in preventive medicine of incalculable value to the state or community, but the lesson has not been learned. According to General Wood the discovery of the transmission of yellow fever resulted in the saving of more lives each year than were lost in the entire Cuban war. The reduction of the death-rate in New York City from 20 per 1,000 in 1903 to 18.75 per 1,000 meant the saving of 4,500 lives, the prevention of 10,000 cases of sickness. It saved the work of one or two great hospitals, it saved some wives from becoming widows, some children from being fatherless and it prevented some poverty.

The installation of a filtration plant costing $7,000,000 in Pittsburg has already decreased the number of deaths of typhoid fever by 100 annually. If a life is worth $5,000 there has already been a saving of $500.000 worth of human life a year, but according to Sedgwick polluted water by lowering the resistance of the organism is also responsible for two other deaths (pneumonia, bronchitis, malnutrition) for every death from typhoid, so that for every 100 lives saved from typhoid, 200 lives are saved from other diseases; therefore $1,500,000 are saved in the year. Had Philadelphia in 1891-92 expended $7,000,000 for a filtration plant it would have saved about $22,000,000 in loss to hotel keepers, merchants, manufacturers, for the care of the sick, loss of time, expense of burial from a typhoid fever epidemic which it had on its hands.

As a nation we have no comprehensive health organisation, our only federal guardian being the Marine Hospital and Public Health service which, to prove that wealth comes before health, is under the direction of the Secretary of the Treasury. The American Health League and the American Medical Association are making a determined effort to have the government exert more paternalism regarding questions of health. It is proposed that a department be created in the cabinet to include the various departments of health now scattered promiscuously throughout various bureaus. The great contending parties have recognised this question and have introduced planks in their respective platforms. With a stimulus of sufficient force from the various medical bodies of the country, we may live to see this praiseworthy reform, but we have a Herculean task to perform in order to overcome the nearsightedness and the penny-wise-and-pound-foolishness of our lawmakers in continually grinding down appropriations for the good of the health of the community. We must awaken a social consciousness and responsibility in matters pertaining to health and sanitation. We must teach that there is an equality in matters of health as is now recognised in matters of law and that it is the duty of the government, federal, state and municipal, to bring about conditions of equality as to health. Why should the death-rate of the poorest class of workers be three and a half times as great as among the well to do? Nature demands that each and every being have pure air, pure water, and pure food and the government must enforce nature's law.

Samuel Hopkins Adams (*McClure's Magazine*, July, 1903), says that but one fourth of the State Boards of Health are actively efficient, the rest being honorary and ornamental. They are willing to work but have no appropriations with which to work. What an economical bearing state work may be is illustrated in the State of Massachusetts where by the issuance of antitoxin and vaccine the citizens of the state saved $210,000 or $74,000 more than the total appropriation of the department. I have previously shown what an enlightened health department, such an one as exists in greater New York or Buffalo, may do for efficient government of a city; through its sanitary bureau in the inspection of lodging houses, tenements, slaughter houses, theatres, through the compulsory notification of contagious diseases and hospitals for the segregation and treatment of same; through the inspection of foods and drugs; through fumigation; by the distribution of literature concerning diseases; also of antitoxin and by the work of its laboratories in the analysis of foods, milk, water, and the like.

It has come to pass that the freedom of an individual to neglect his own health and injure that of others is being gradually eliminated in the United States. Aside from his vocation in the diagnosis and treatment of disease, the physician must also be an educator because he forces people to supplant ignorance with intelligence, to elevate the ideals of life, and to force the conduct of society into new avenues of activity.

There are conditions which sap the vitality of a nation arising from individual errors and which of all others are potent for evil. They depend upon errors of individual life which multiplied by intuition and training and ignorance become the self-inflicted wounds of a nation. We must therefore seek to control the individual unit by an education of the masses. In this work we are confronted by the great obstruction of ignorance and superstition for the masses often receive twentieth century truths with receptive minds of the tenth century. The discourses of the bacteriologist and pathologist have little more than academic interest unless we can educate the masses. The medical profession has been taught the value of this course in the successful war now being waged against the Great White Plague. In this disease we are so far successful because we have thrown away the sham of medical exclusiveness and have made an alliance with nursing associations, social workers, clergymen, educators and philanthropists and thus, by similar methods, other preventable diseases may be conquered.

The mortality of infant life is appalling. It is due to the ignorance of the mother and is greatest in proportion to the condition of poverty and the density of population. It has been designated as the "slaughter of the innocents" and still by educating the mother in cleanliness and the proper feeding of babies many an infant can be saved. "A helpless infant is entitled to milk that gives, not takes life." Dr. George Goler, health commissioner of Rochester, N. Y., a graduate of this college, has reduced infant mortality in Rochester to such an extent as to call forth the admiration of the country, by having the municipality establish milk stations in that city for the free distribution of proper milk to infants. Nathan Strauss, through the establishment of his milk stations in New York city and abroad has proven himself one of the useful members of our civilisation.

The newspaper press has done much to hinder the progress of medicine in the past by its selfish and unethical alliance with purveyors of nostrums and quacks and by championing the cause of the misguided, such as antivaccinationists, antivivisectionists, and the like, but a new service is open to it in spreading into the homes of the poor and ignorant the gospel of truth in health matters; and so the school and the college must assist, and I am glad to note that a school of sanitary science will open its first session at Cornell University this year. Louisiana has a system of hygienic education by which hygienic institutes are held under the authority of the State Board of Health. These institutes are held before colleges, and high schools; by this means a great number of the populace are instructed in the prophylaxis and nature of transmissible diseases. The movement is extended by the perfect correlation of the clergy of all denominations, educators, and educated persons with the health service of the state.

The schools should teach not only hygiene, and physiology, but social science and economics and the benefits to a nation of a moral and religious family life. As an example of this assertion, we would not need to concern ourselves with the question of alcoholism in its degredation of human kind and its production of poverty, if the hygiene of the body and the mind and the value of the soberness of life were taught. Richardson says, "If you ask what has produced such a marvelous vitality in the Jewish race, the answer is not due to a particular physique, but mainly from the necessities of his persecuted career that he has retained his vital capacity. In a word, he has been forced into soberness of life. His home was made more exclusive to him than to freer man and became the simple center of his earthly career. Thus, he was more temperate, more chaste, more attached and considerate to his enfeebled young and old of that center, hence more careful of tomorrow."

The doctor (meaning one qualified to teach—Webster), is therefore a minister, for his is the function to change the belief from a lower to a higher form, and to bring the conduct of society into the subordination of belief (Blackmar). As education will do good in the examples which I have described, there still is another example which occurs to me where it will prove of value. It is said that neurasthenia is a disease of the rich but it is equally encountered among the poor. When you consider the continuous daily grind of factory work, feeding a machine in the same manner day after day, there is little wonder that the brain under this never changing toil chafes and that the toiler becomes irritable and possessed of obsessions. Education, explanation, and advice will do more good than all medicines and we owe Du Bois much thanks for teaching us the value of good sensible talk.

From a sociological view point, perhaps, one of the most prolific sources of disease and crime has been the result of herding large numbers of families in tenements. Up to the time of the Tenement House Commission and the enactment of proper laws regulating the tenements, some of these houses were so thoroughly infected with vermin and filth that destruction seemed the only remedy. Some years ago, the municipality of Glasgow by the destruction of such houses reduced the death-rate from 55 per 1,000 to 14 per 1,000. It is estimated that in the tenements of New York city there are 370,000 dark rooms. People are thus deprived through no fault of their own but through errors of living and the greed of landlords, of fresh air, and of sunlight, with the result of terrible infant mortality, consumption, bronchitis, pneumonia and a greater evil, that of crime and of prostitution and a sinking of the morals. Hunter says, "You rob children through the yardless tenement of play, their first and only occupation, and send them upon the street to learn vagabondage and prositution, just because a community is selfish and thoughtless." We owe everlasting gratitude to Jacob Riis, that excellent sociologist and philanthropist, New York's most useful citizen, who has studied the pathology of mankind and has prescribed radical cures, for demonstrating in the great city of New York that it is better to spend money for playgrounds than it is to enlarge the reformatories and state's prisons.

In the fight against the unsanitary tenement, it is fortunate that Buffalo has a health commissioner who against heavy odds and political intrigues does not fear to hail the unconscious owner of the tenement into court nor to turn off the water supply until his recommendations are obeyed. It is true that individuals and owners of foul tenements resent by all legal means the elimination of the perilous conditions from which they make their profit.

There are questions which concern the physician and where he is called upon as an overseer in regard to education. The school is the only place where it is expected that inequalities and handicaps of home environment shall be obvious. It is the duty of the physician to determine whether the curriculum is too burdensome, whether the school-room is properly ventilated, whether the children attending the school are undernourished or rickety. whether or not they suffer from physical defects such as enlarged tonsils, astigmatism, and the like. Are there in our schools as in New York city 66 per cent. in serious need of medical dental or ocular care? Does the condition exist because of ignorance of the parent or neglect or indifference, or because of insufficient income? Children in parochial and public schools have the right to be examined and told their deficiencies and how to correct them.

Allen, in *Efficient Democracy,* says, "simplified breathing is more important than simplified spelling. They must be taught that a nose plus adenoids makes backwardness, that decayed teeth times ten equals malnutrition, that hypertrophied tonsils are infinitely more menacing than hypertrophied playfulness." Buffalo, I am glad to say, has this year for the first time instituted the medical inspection of schools. I have but one suggestion further to make, and that is that the municipality feed the undernourished children of school age. This is considered by such a writer as Spargo of more importance than the medical school inspection. The child of the inebriate, of the idler, and of the criminal must not be made to suffer for his parents' sin.

The state is often called upon to support at public expense as paupers the men, women and children brought into poverty through industrial conditions. Instead of building almshouses and lodging houses it were better to enact laws for industrial compulsory insurance against distress, disease, accident, and death, as now practised in Germany, or pensions for those workers who have reached 70 as now practised in England. "There is no other nation," says Hunter, "comparable industrially to the United States which is so backward in its knowledge, in its legislation, in its administrative machinery for dealing with unsanitary conditions in factories, mines, workshops, and in preventing those dangerous processes in industry responsible for so large a number of unnecessary diseases and accidents and deaths."

President Roosevelt, by the creation of a Homes Commission of which Brigadier General Sternberg is chairman and Professor George M. Kober, M. D., is secretary, has done an excellent service. As chairman of a subcommittee on social betterment Kober has recently published a noteworthy contribution on Industrial Hygiene. A list of occupations and trades dangerous to health is given, for it is desirable to know the relation of a person's trade to his health, the source and significance of the dangers and the possible means for the prevention and mitigation of their injurious effects. In the manufacturing and mechanical industries, for instance, millers, coopers, brewers, distillers and rectifiers, cigar makers, tobacco workers, painters, glaziers, varnishers, marble and stone cutters are dangerous occupations; indoor work and work involving exposure to irritating dust, poisonous vapors, extremes of heat and cold and abnormal atmospheric pressure are dangerous to health.

It is interesting that in this country, the first law as to safety and sanitation in factories was passed in 1877, since which time all the states and territories have enacted some form of labor or factory law. Children should be advised not to choose occupations inimical to health until such a time that they are thoroughly safeguarded. Is there any reason why consumption should claim 48.13 per cent. of the deaths of printers unless it be that the work is performed in an impure and dusty environment? Children should be also warned against those occupations in which the use of alcohol prevails and to seek outdoor rather than indoor work such as the sweat shops offer. Child labor, however, is a menace to morals and good citizenship. In the days prior to the invention of machinery, child labor was performed, but always under the eye of the parent and in the healthful home of the hamlet. This was the era of craftsmanship and this in a measure was educative. Child labor as now conducted from a national point of view is a waste of its most valuable asset—manhood.

With Hood we exclaim: "Oh, God, that bread should be so dear and flesh and blood so cheap."

According to the census of 1900 there were 1,752,187 children under the age of 16 years employed in gainful occupations. In the state of New York there were 76,000 children out of school in the twelve months covered by the census. The textile industries rank first in the enslavement of children. In the South about 20,000 are thus engaged while in the United States 80,000 are employed. In the manufacture of glass 13.45 per cent. of the total employed are children under 16. There are 25,000 boys employed about the mines as possible recruits for a future army of asthmatics and consumptives. There are 12,000 employed in the cigar and tobacco factories, 10,000 in wood-working industries and equal numbers in laundries, messenger boy service, canning factories and the like.

Females suffer by reason of factory life and this is true among the unmarried women. The Committee of fifteen speaks as follows: "It is a sad and humiliating admission to make at the opening of the twentieth century, in one of the greatest centers of civilisation that in numerous instances it is not passion or corrupt inclination but the force of actual physical want, that imperil young women along the road to ruin."

They urge as one of the fundamental policies to combat the social evil, the improvement of the material condition of the wage-earning class but especially of the young wage-earning women. But factory life is especially demoralising in married women because their houses are ill kept, their children neglected and their infants which should be at the breast improperly fed and nourished. Overwork and fatigue are predisposing causes to disease because of the accumulation of waste products in the blood and the wear and tear on the nervous system, both of which make the body more susceptible to disease. Thus, long hours and tedious work not only endanger the individual life but the many railroad accidents and deaths therefrom may be attributed to this cause which led the United States in passing laws regulating the hours of railroad employees.

As to the importance of sanitation in work shops, little need be said. New York state allows thirty-three distinct industries to be carried on in living rooms and there are 23,000 such factories in New York city alone. Dr. Annie S. Daniels says, "Every garment worn by a woman is found being manufactured in tenement rooms. This is also true with reference to articles worn by infants, also such articles as furs, cigars, pocketbooks, paper boxes, and the like. Often such articles are handled and stored in infected rooms." Of 150 families examined by Dr. Daniels, sixty-six continued at work during the entire course of the contagious disease, for which she attended the families. Could a better example be provided for the necessity of compulsory notification of contagious diseases and the need of a municipal contagious hospital? Basenquet (quoted from Richmond The Good Neighbor) says, "If we will have nasty things, shoddy things, vulgar things, we are condemning somebody to make them. If we will have impossibly cheap things· we are condemning somebody to work without pay," and so another body of laymen and laywomen is formed to overcome these evils. The Consumers League urges reforms in that the great department stores in their treatment of their employees, give them employment upon such terms as to enable the employee to lead a selfrespecting and moral life, and urges the purchaser to ask only for such underwear made under sanitary conditions and without child labor or sweat shop labor.

One of the reforms created during President Roosevelt's administration is the enactment of the Food and Drugs act of June 30, 1906. It was through the untiring work of the chief of the United States Bureau of Chemistry, Dr. Harvey W. Wiley, that these laws were enacted. But public opinion, which was lukewarm, was aroused through Upton Sinclaire's book "The Jungle," which caused the passage of this law by an almost unanimous vote. The object of this act is to prohibit the interstate traffic in adulterated and misbranded food products and to forbid the manufacture and sale of such foods and drugs in the United States. The competition of trade and desire after gain led manufacturers and merchants to debase the quality of goods without changing their names. For instance, in the great slaughtering industry of the country it was found that oily and fatty parts of animals were worth less in the ordinary state than in the fat of milk when manufactured into butter, so that these fats were so treated as to sell for butter and in the same manner drugs and chemicals masqueraded under false names, as alum in baking powder and drugs injurious to health were added to foods as preservatives, all of which were injuring greatly the health of communities and especially the poorer people because of their inability to buy goods of better quality.

Since this law became effective, the products manufactured are vastly superior to what they were before. The habit forming drugs as alcohol, alpha and beta eucaine, opium, cocaine, morphine, chloral hydrate, cannibis indica and acetanilid can only be sold in pharmaceutical preparations when their name is plainly marked upon the labels, thus hundreds of nostrums which were formerly sold indiscriminately through flaring newspaper advertisements to the public such as Peruna and similar nostrums had their nature revealed to the consumer. There will be fewer drug habits formed in the future than in the past. With the education the public is now receiving on the alcohol question, such as an article, "Alcohol and the Individual," by Dr. Harry Smith Williams, in October McClure's, it will not be many years before the hygiene of the body and the mind will overcome the necessity of the state control of this and similar traffics, and for the same reason the venereal peril will not have the danger which is now so apparent.

The lesson must be brought home to the people, that they are responsible for much of the disease and suffering, and so it is now important to study the relationship of home surroundings on disease.

Laws may decree the erection of sanitary dwellings, but these in themselves are of no avail unless the tenant observes ordinary rules of cleanliness and ventilation. A bathtub may be useful if the coal supply is stored in it, but the health of the community were better served if it were used for the purpose for which it was intended. According to Dr. Richard C. Cabot, in our dispensary and hospital we have not done all the efficient work possible because we merely located disease. "We take accurate aim but do not fire when we aim." Hence in every dispensary and hospital there should be a social service department working in conjunction and under the supervision of the physician to scatter and dislodge disease so accurately located by our diagnostic field glasses. It is now imperative in such diseases as tuberculosis and neurasthenia to fight the disease not only by drugs, but by pervention, education, and if need be by financial help in the home. Thus we call upon the nurse, the educator, the settlement worker, trained in the diagnosis and treatment of character under adversity, ministers and philanthropists to assist us.

Since October, 1906, there has been a social service department in Massachusetts General Hospital, efficient as will be seen by the fact that 2,124 cases were treated in 1905-1907, and under the direction of that excellent and useful citizen Richard C. Cabot of Boston. So successful has the work proven itself that similar departments have been started in the Johns Hopkins Hospital, Baltimore, and in Chicago. In Johns Hopkins the students have taken an interest in this service and it would be well if a similar service were to be started among the students here. The object of this work goes beyond the consultation in the dispensary or the ward of the hospital. It seeks change in diet, in environment and in general reform of the home life. The social worker acts as intermediary between the patients and the different organisations of the city which can be used for his welfare, and this field of work offers to physicians and students an excellent opportunity to learn through practical demonstration the value of sociologic knowledge in the field of medicine. Two years ago Dr. Theodore C. Janeway established a bureau for those handicapped by disease, such as the loss of an arm, by phthisis, rheumatism, deafness, and the like. Some persons entirely unfit for certain work by reason of physical defect may be useful in some other position.

No hospital inmate should be discharged until, through investigation it is found that his treatment can be followed up at home. It would be praiseworthy if some person in Buffalo, charitably inclined, would found a convalescent home where those discharged from the wards of hospitals could continue the advantage of treatment received at the hospital. The need of a link between the hospital and the dispensary and the patient's home and environments is obvious and has been in a measure supplied through the establishment of social service departments in Boston and elsewhere

There are many important subjects which suggest themselves which I have omitted from discussion in this paper for lack of time, such as homes for defectives, idiots, imbeciles, epileptics and the insane, the treatment of the criminal, creches for the children of the poor working women, fresh air missions and gymnasiums for the reëducation of tabetics, spastic paralytics, and forest schools for the undernourished. It is a pleasure to me to have had this opportunity to call to your attention the relation of sociology to medicine in its present needs and future problems. It is especially gratifying because Buffalo by the establishment of the first Charity Organisation in the United States in 1877, was the first city to apply philanthropy scientifically, in that in dealing with the poor it relieved not only the present want but also found out and removed the causes of that want and restored to society persons thus assisted. So our work is not only to cure present disease, but to prevent it by overcoming all the conditions and many of the bad environments which nourish it. It is a social service and demands more than any other vocation a knowledge of all sides of human thinking, feeling, and acting. It is combined with politics, with economy, with psychology and with ethics. It is a work of the brain and of the heart, and thus in closing I quote Burns:

"Then let us pray that come it may,
  As come it will for a' that
That sense and worth, o'er the earth,
  May bear 'the gree' and a' that,
For a' that, and a' that; it's coming yet, for a' that;
  When man to man, the world o'er
Shall brother be for a' that."

400 Franklin Street.

